# The burden of psychological distress in stroke survivors: a systematic review and meta-analysis of sociodemographic and behavioral factors

**DOI:** 10.3389/fpubh.2026.1930488

**Published:** 2026-09-28

**Authors:** Jiali Chen, Yihao Wang, Ziyu Zhang

**Affiliations:** 1 Department of Global Medical Research Promotion, Shinshu University Graduate School of Medicine, Matsumoto, Japan; 2 Luoning County Health Commission, Luoyang, Henan, China; 3 Department of Geriatrics II, Aerospace Center Hospital, Beijing, China

**Keywords:** mental health, meta-analysis, psychological distress, social support, stroke, systematic review

## Abstract

**Background:**

Psychological distress is a common yet frequently overlooked complication among stroke survivors, significantly impacting their recovery and long-term prognosis. Identifying the prevalence and key psychosocial determinants of this condition is essential for developing effective mental health support strategies. However, the existing evidence remains fragmented. Therefore, a systematic review and meta-analysis are urgently required to consolidate current evidence on the prevalence and risk factors of psychological distress in stroke survivors. This synthesis will serve as a pivotal reference for optimizing clinical management and shaping future healthcare policies.

**Objective:**

To systematically evaluate the prevalence of psychological distress among stroke survivors and identify the associated sociodemographic and psychosocial risk factors.

**Methods:**

Eight databases were systematically searched for observational studies from January 2019 to February 2026. The search terms were “Stroke” “Psychological Distress” and “Emotional Distress.” The Agency for Healthcare Research and Quality (AHRQ) was used to evaluate the study quality, and the meta-analysis was performed using R4.3.2 software.

**Results:**

A total of 22 observational studies involving 15,370 stroke survivors were included in this review. The pooled prevalence of psychological distress was 51.97% [95% CI: 37.79, 66.15%]. Meta-analysis revealed that protective factors against distress included receiving social support (r = −0.37, *p* < 0.0001), the utilization of effective coping strategies (r = −0.47, *p* < 0.0001), and having a spouse (OR = 0.78, *p* = 0.0255). Conversely, higher levels of distress were significantly associated with lower income (OR = 6.69, *p* = 0.0044) and increased levels of disease-related stigma (r = 0.61, *p* < 0.0001). Furthermore, maladaptive coping mechanisms, such as avoidance (r = 0.48, *p* < 0.0001) and submission confrontation styles (r = 0.61, *p* < 0.0001), were identified as significant contributors to psychological morbidity.

**Conclusion:**

Psychological distress is highly prevalent among stroke survivors. Our findings identify social support, marital status, income, and coping mechanisms as pivotal determinants of psychological distress in stroke survivors. These results underscore the necessity of integrating targeted, multidisciplinary interventions into routine post-stroke care to alleviate distress and enhance long-term quality of life.

**Systematic review registration:**

Identifier https://www.crd.york.ac.uk/PROSPERO/view/CRD42024570838.

## 1 Introduction

Stroke is a neurological deficit resulting from an interruption or reduction in the blood supply to the brain (1). Stroke remains the second-leading cause of death and the third-leading cause of death and disability combined (2). Furthermore, the absolute number of cases increased substantially from 1990 to 2019, representing a significant global health burden (3–5). The emergence and development of stroke is influenced by a number of factors, including core health behaviors (such as smoking, physical activity, diet and weight) and health factors (such as cholesterol, blood pressure and glucose control) (6–9). Additionally, according to the World Stroke Organization, with the advancement of medical technology, the mortality rate of stroke is continuously declining, and an increasing number of people are surviving stroke events, which poses a significant challenge in the management of stroke survivors (10).

Mental health issues represent a significant challenge in stroke survivors. Psychological distress is a subjective experience that arises primarily from unmet psychological needs (11). National Comprehensive Cancer Network (NCCN) defines it as a continuous process ranging from normal psychological activities such as emotional vulnerability, sadness, and fear to psychological disorders such as anxiety, depression, panic, spiritual crisis, and social loneliness. It is characterized by the perception of negative changes in the self and its functioning, accompanied by intense negative emotions. It is a prevalent complication which frequently affects the mental state and quality of life of patients with a variety of conditions, including non-small cell lung cancer patients (12), head and neck cancer survivors (13), brain tumor survivors (14), intensive care unit survivors (15), chronic kidney disease (CKD) patients (16) and stroke patients (17). It is a common occurrence following a stroke and has an adverse impact on recovery (18). This can subsequently result in considerable economic and psychological burdens on society and families.

Stroke survivors frequently experience a constellation of physical and sensory impairments—including motor deficits, vestibular dysfunction, sleep disturbances, and chronic pain—which collectively compromise self-care independence and vocational capacity. These functional limitations, coupled with the strain of altered social roles and the substantial economic burden of long-term rehabilitation, exert a profound negative impact on the mental health of this population (19). Therefore, it is recommended to proactively investigate the potential influencing factors of psychological distress and implement timely intervention measures, in order to reduce the recurrence rate of the disease and facilitate the recovery process.

Although several systematic reviews and meta-analyses have reported on the prevalence and correlates of psychological distress among stroke survivors, the results vary depending on the setting and instruments used. This makes them difficult to compare. In particular, differences in recruitment (e.g., stroke type), outcome measures [e.g. insomnia (38.2%) (20), fatigue (48%) (21) and depression (27%) (22)] and assessment tools (e.g., the Pittsburgh Sleep Quality Index [PSQI] and the Hospital Anxiety and Depression Scale [HADS]) may contribute to heterogeneity in prevalence estimates Therefore, a systematic review and meta-analysis are warranted to derive pooled estimates and to explore sources of heterogeneity.

The impact of psychological distress (e.g., depressive and anxiety symptoms, and related mental health outcomes) on stroke patients has been investigated (23–25). However, the evidence base remains fragmented and prior reviews have often focused on a single psychological indicator or failed to provide a comprehensive synthesis of psychological distress and its associated risk factors. This study aimed to address these gaps by systematically evaluating and meta-analyzing the prevalence and incidence of psychological distress among stroke survivors, and synthesizing associated risk factors. By pooling existing evidence, we aimed to derive a more precise overall estimate of psychological distress, and reconcile inconsistent findings regarding its correlates reported in individual studies and earlier reviews. The resulting evidence will inform future research and support more targeted clinical strategies for mental health screening and intervention in stroke care.

## 2 Methods

The study was conducted in accordance with the Preferred Reporting Items for Systematic Reviews and Meta-Analyses (PRISMA) guidelines and was also registered on PROSPER (registration number: CRD42024570838).

### 2.1 Search strategy

The researchers conducted separate searches for English and Simplified Chinese studies related to the association between the psychological distress and stroke survivors. A literature search was performed in PubMed (*n* = 212), Cochrane Library (*n* = 9), Embase (*n* = 510), Chinese National Knowledge Infrastructure (CNKI) (*n* = 230), Wan Fang Data (*n* = 35), Chinese Science and Technology Journal Full-Text Database (VIP) (*n* = 32), and Foreign Medical Literature Retrieval Service (FMRS) (*n* = 154) from Shenzhen METSTR Technology Co., Ltd., until February 2026. The search terms were “Stroke” “Psychological Distress” and “Emotional Distress.” Additionally, the reference lists of relevant publications were manually examined to uncover further studies. A detailed search strategy for PubMed was shown in Supplementary Table 1.

### 2.2 Eligibility criteria (inclusion)

The present study incorporated observational studies (case–control, cohort, or longitudinal designs) that recruited adult stroke patients (aged ≥18 years) and reported data on significant psychological distress among stroke survivors, as defined by the original studies, using validated instruments and/or predetermined cut-offs. For the purpose of synthesis, studies were required to report the number and/or proportion of participants classified as experiencing significant psychological distress. For the purpose of associated-factor synthesis, studies were required to report extractable effect estimates (e.g., odds ratios/risk ratios/regression coefficients) for factors associated with significant psychological distress in stroke patients.

### 2.3 Eligibility criteria (exclusion)

Studies were excluded if full text was inaccessible, if no relevant outcomes were reported, if the study design was unclear, or if the same study was duplicated. Decision rule for extractable primary outcome: We included only studies that reported the number and/or proportion of adult stroke survivors who met the study-defined threshold for significant psychological distress using validated instruments (e.g., K10, DT, HADS, GSI, K6). Studies that reported only continuous symptom scores without a threshold-based classification (and thus without extractable number/proportion) were excluded.

### 2.4 Study selection

The search results were imported into NoteExpress software. Two researchers (CJL and WYH) independently screened the studies using the aforementioned inclusion criteria. Any discrepancies between the two researchers were resolved through discussion with a third researcher (ZZY). After removing duplicate studies, the two researchers screened the titles and abstracts to exclude studies that did not meet the criteria. They then read the full article to identify the most relevant research. Additionally, the list of references included in the studies was used to search for further relevant studies.

### 2.5 Data extraction

Data extraction was conducted independently by two reviewers (CJL and WYH) using a pre-designed, bespoke data extraction table. The extracted information included population characteristics, exposure factors, comparisons, outcomes (PICO elements) and study methods that were relevant to the review question and specific objectives. The extracted data were cross-checked through a consensus process between the two reviewers. Any discrepancies were resolved through discussion and mutual agreement. If consensus could not be reached, a third reviewer (ZZY) was available to adjudicate and make the final decision. The data extraction table was not piloted on a separate subset of studies prior to full extraction.

### 2.6 Risk of bias assessment

The quality of the studies was assessed using the Agency for Healthcare Research and Quality (AHRQ), recommended quality assessment criteria for cross-sectional studies, which include 11 items. These items are used to evaluate the quality of the study and are rated as “yes,” “no,” or “unclear.” The scoring standard is as follows: “yes” is scored as 1 point, while “no” and “unclear” are scored as 0 points, with a total of 11 points possible. Based on the total score, the quality of the study is divided into three levels: 0–3 points for low quality, 4–7 points for moderate quality, and 8–11 points for high quality. This assessment standard is widely used to evaluate the quality of cross-sectional studies to ensure the reliability and validity of the study results (26). Quality assessment was conducted independently by two reviewers (CJL and WYH). Disagreements were resolved through discussion, and when consensus could not be reached, a third reviewer (ZZY) made the final decision.

### 2.7 Statistical analysis

In this study, the “meta” and “metafor” packages of the R4.3.2 software were employed for the purpose of achieving the desired statistical analysis of the data. A heterogeneity test was conducted using both the *I^2^* and Q tests. The choice between fixed-effects and random-effects models was based on between-study heterogeneity. Fixed-effects models were used when heterogeneity was low (I^2^ < 50% and *p* ≥ 0.05); otherwise, random-effects models were used. The results of the meta-analysis were presented using a forest plot. Publication bias across studies was assessed by visually inspecting funnel plots, and its potential impact was further evaluated using the trim-and-fill method. Sensitivity analyses were performed using the leave-one-out method, and inter-study heterogeneity was explored using meta-regression. Statistical significance was set at *p* = 0.05 (two-sided), and potential differences in effect sizes across included studies were considered. Data transformation was conducted as much as possible to accommodate between-study discrepancies. If the original studies reported the number and/or proportion of participants, odds ratios (ORs) were used directly as the effect size in the pooled analyses. If the effect size was Pearson’s correlation coefficient (r), the standard error (SE) was calculated as $\mathrm{SE}(r)=\sqrt{\frac{1-r^{2}}{n-2}}$ (*n* = the sample size, r = the correlation coefficient). Standard errors of the correlation coefficients were calculated and combined analyses were performed using r as the effect size; If the effect size was reported as an OR, the following formula was used to derive the corresponding standard error: $\mathrm{SE}(\text{OR})=\frac{\text{uperCI}-\text{downCI}}{2Z}$ [where uperCI is the upper limit of the confidence interval for the OR, downCI is the lower limit of the confidence interval, and Z is the Z-score for a normal distribution, which is 1.96 for a 95% confidence interval (*CI*)] in order to ascertain the requisite SE value. The OR estimates were then used in the statistical analyses of the pooled effect size.

## 3 Results

### 3.1 Selection outcomes

Upon conducting a database search, a total of 1,182 articles were retrieved. After applying the inclusion criteria, 22 studies were deemed suitable for inclusion in the systematic review and meta-analysis. The study selection process is presented in Figure 1.

**Figure 1:**
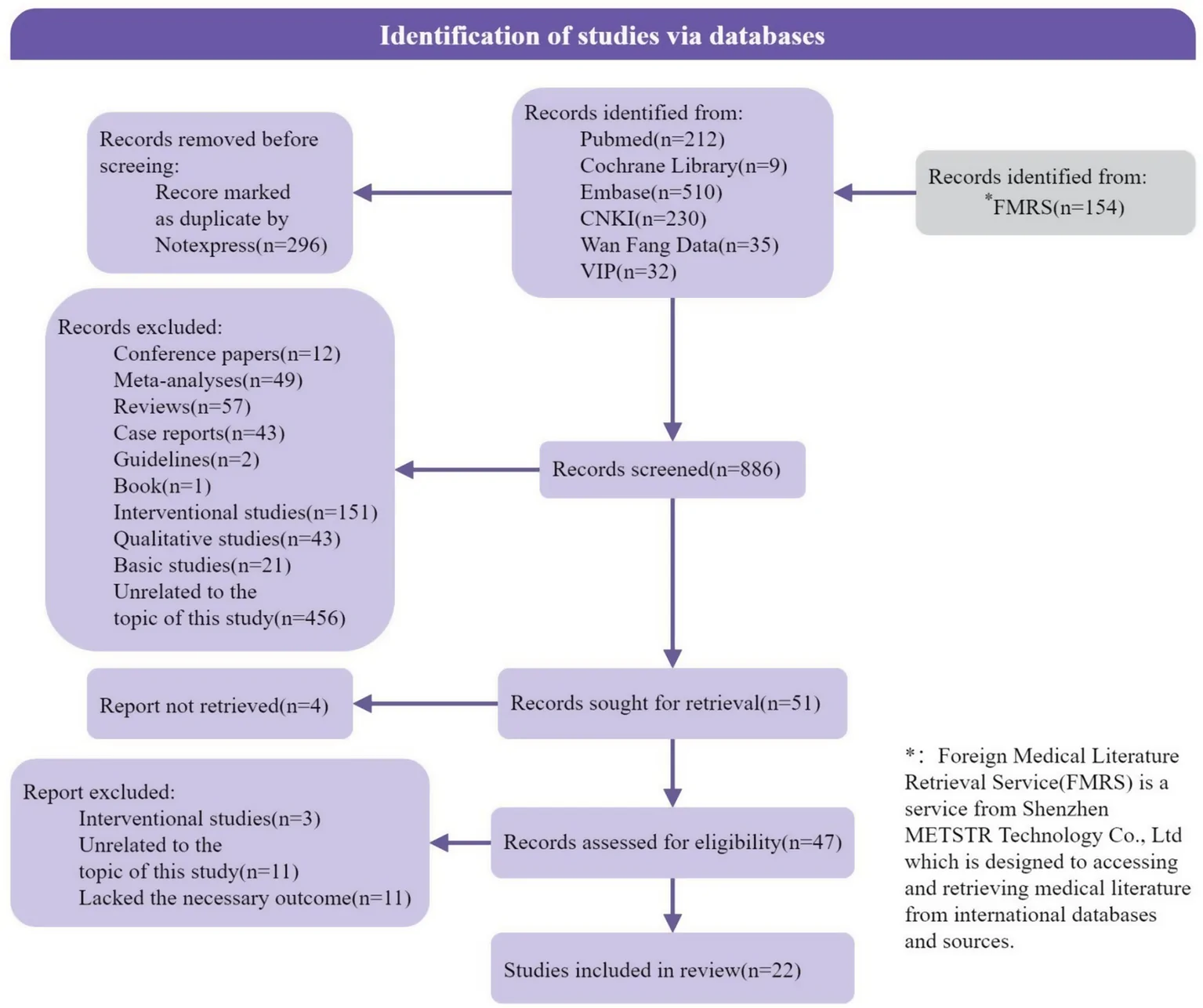
Literature screening process flowchart.

### 3.2 Study characteristics

The included studies primarily comprised 15,370 stroke survivors from various locations, including Zhoushan, Tangshan, Zhengzhou, Yinchuan, Nanchang, Luzhou, Zhangzhou, Guangzhou, Xuzhou, Xi’an, Tianjin in China, Verona in Italy, Kinshasa in the Democratic Republic of the Congo, Colombo in Sri Lanka, and the United States of America (27–48). One of the included studies, Xuan D (48), was not included in the meta-analysis because it did not provide extractable threshold-based data on the number (and/or proportion) of participants with significant psychological distress; therefore, it could not contribute to the pooled estimates for associated factors. The fundamental characteristics of the included studies and the associated factors involved in each study are presented in Table 1. It presents the incidence of psychological distress, sample size, survey locations, various scales and tools used to define psychological distress in each study, AHRQ quality assessment scores, as well as different associated factors and their significance. From this, we can see that the AHRQ scores of the studies all fall within the range of 4–6 points, indicating that they are of moderate quality. At the same time, there are significant differences in the assessment tools used and the incidence of distress across the studies.

**Table 1:** Basic information of the included studies.

Study	Countries and regions	Evaluation criteria	Sample size	AHRQ score	Incidence rate	Associated factors
Zhu et al. (45)	Zhou Shan, China	k10 ≥ 16	125	4	61.60%	1,2,3,6,7,8,9,10,11*,12*
Zhu et al. (32)	Tang Shan, China	DT ≥ 5	203	4	58.13%	13*,14*
Zhang and Xiong (44)	Nan Chang, China	DT ≥ 4	200	5	68.50%	15*
Zhang and Li (38)	Zheng Zhou, China	DT ≥ 4	200	6	62.00%	1*,4*,6*,16*,17*
Zhang and Cui (43)	Zheng Zhou, China	DT ≥ 4	242	5	52.07%	1*,2,3*,4,5*,6*,9,18,19*,20*,21*,22*,23*,24*
Liu et al. (42)	Zheng Zhou, China	k10 > 15	381	5	58.27%	2,5*,16*,25*,26*,27*,28,29*
Liu et al. (36)	Lu Zhou, China	DT ≥ 5	258	5	53.10%	2*,3,4,5*,6,7*,8,16*,26,30*,31*,32*,36*
Liu et al. (41)	Lu Zhou, China	DT ≥ 5	330	6	62.12%	1,2,5,6,7,9*,20,22*,30*,33*,34*
Li et al. (31)	Tang Shan, China	DT ≥ 5	335	6	67.16%	1*,2,3,5*,6*,7*,16,18*,21*,23*
Li et al. (30)	Tang Shan, China	DT ≥ 5	467	6	67.88%	1*,2*,3,5*,6*,9,18*,20
Li et al. (40)	Zhang Zhou, China	DT ≥ 4	165	5	69.70%	
Gu et al. (28)	Guang Zhou, China	DT ≥ 4	288	6	71.53%	1,2*,3,5*,6*,7,8,18,22*,23*,30*,35
Bi and Chen (39)	Xu Zhou, China	k10 > 40	80	4	31.25%	36*,37*
Liu (37)	Xi An, China	HADS≥11	586	6	61.09%	
Li (29)	Tang Shan, China	DT ≥ 5	716	4	52.65%	1*,2*,3,5*,6*,8*,16,18*
Gandolfi et al. (27)	Verona, Italy	GSI > 0.57	50	5	26.00%	1,2,3,4,6*,9*,19,31*,33,38,39
Dong et al. (33)	America	K6 ≥ 13	9,746	6	13.08%	1,2,3,5*,8*,30*,40*
Manzekele Bin Kitoko et al. (34)	Kinshasa, The Democratic Republic of the Congo	HADS>14	85	5	27.06%	2,3,6*
Weerasekara et al. (35)	Colombo, Sri Lanka	K10 ≥ 22	177	4	23.16%	1*,2*,7*,10*,16,30*,41*,42*
Lei et al. (47)	Tian Jin, China	DT ≥ 5	447	4		1*,3*,43*,18*4*,5*,16*,9,33,20,30,31*,23*,21*,44*
Duan et al. (46)	Zheng Zhou, China	k10	86	4		1,2*,3*,5,6,8*,23*,36*,45*
Du et al. (48)	Yin Chuan, China	k10	203	5		22*,46*

k10: 10-item Kessler psychological distress scale; DT: Psychological Distress Thermometer; HADS: Hospital Anxiety and Depression Scale; GSI: Global Severity Index; K6: 6-item Kessler psychological distress scale. 1: Age, 2: Gender, 3: Marital status, 4: Employment, 5: Income, 6: Educational level, 7: First episode; 8: Health insurance, 9: Type of stroke, 10: Family history of anxiety and depression, 11: Stress response, 12: Alexithymia, 13: Rumination, 14: Post-traumatic growth, 15: Perceived burden, 16: Caregiver, 17: Dysphagia, 18: Place of residence, 19: Site of brain injury, 20: Other chronic diseases, 21: Self-efficacy, 22: Coping strategies, 23: Social support, 24: Functional independence, 25: Medical economic burden, 26: Family history of stroke, 27: Disease cognition, 28: modified Rankin Scale, 29: Caregiver family resilience, 30: Self-care ability, 31: Sequelae, 32: Self-acceptance, 33: Disease duration, 34: Timely medical attention, 35: Religious belief, 36: Stigma, 37: Quality of life, 38: Laterality of brain injury, 39: Interest in psychological support, 40: Race/ethnicity, 41: Patient is the sole breadwinner of the family, 42: History of mental illness, 43: Number of children, 44: Level of hope, 45: Psychological resilience, 46: Intimate relationships; *Indicating a significant correlation with psychological distress in the relevant studies.

### 3.3 Meta-analysis results

#### 3.3.1 Stroke survivors’ prevalence of psychological distress

The combination of the 19 studies (*n* = 14,634) (27–45) that reported the number of people with significant psychological distress demonstrated substantial heterogeneity (*p* < 0.001, *I*^2^ = 99.5%). Consequently, the random-effects model was employed, and the combined incidence rate was determined to be 51.97% [37.79, 66.15%] (Figure 2). The sensitivity analysis result was *p* = 0.53 [95% CI: 0.38–0.66], indicating stability (Supplementary Figure A.1). The funnel plot suggested pronounced funnel plot asymmetry consistent with potential publication bias (Supplementary Figure A.2). We therefore conducted the trim-and-fill procedure as a sensitivity analysis to explore how the pooled estimate might change if some studies were missing due to publication bias. Because between-study heterogeneity was present, the trim-and-fill adjustment may be less reliable than when heterogeneity is minimal. The adjusted pooled effect size was 0.23 (95% CI: 0.10–0.35) (Figure 3). To elucidate the source of heterogeneity, meta-regression was employed, whereby the proportion of male survivors, the proportion of low-income individuals, and the mean age were introduced as covariates into the equation. This revealed that the majority of inter-study heterogeneity could be attributed to these covariates. The majority of the between-study heterogeneity could be explained (*QE* test *p* = 0.1139, *I*^2^ = 53.97%, *R*^2^ = 73.63%).

**Figure 2:**
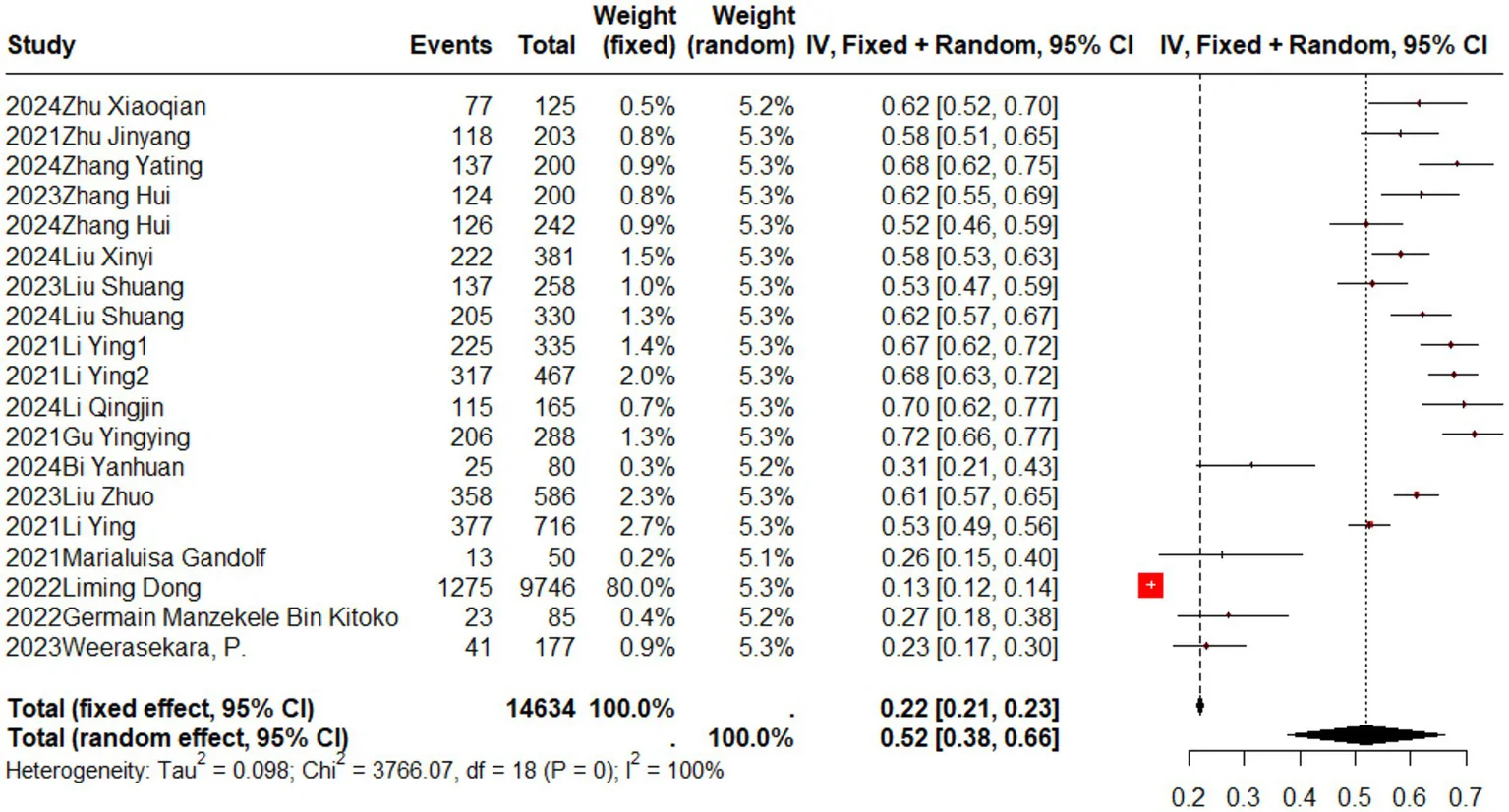
Stroke survivors’ distress incidence forest plot.

**Figure 3:**
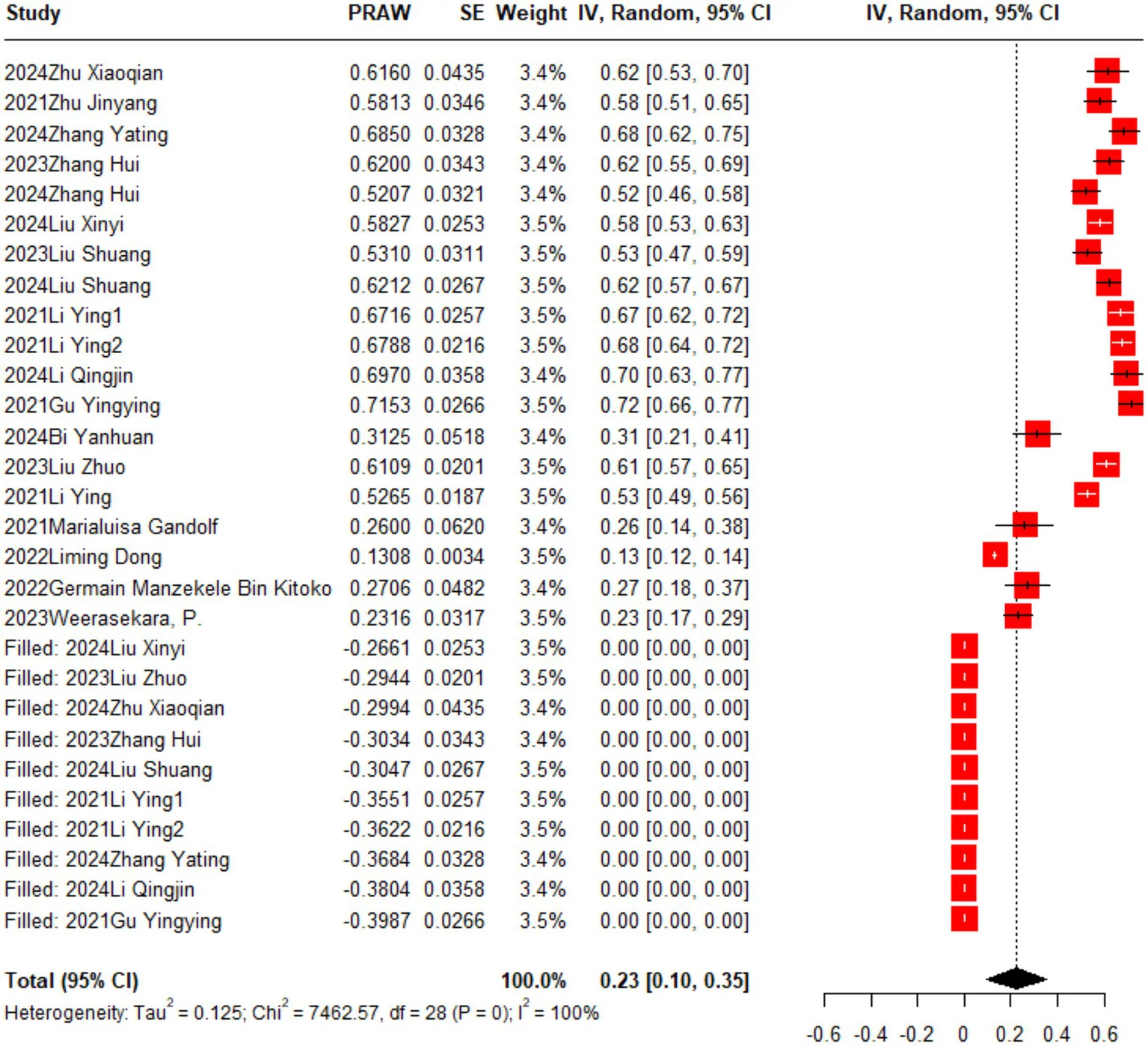
Stroke survivors’ distress incidence trim and filled forest plot.

### 3.4 The factors associated with the occurrence of psychological distress in stroke survivors

The results of the analysis of risk factors associated with stroke survivors are presented in Table 2. Following the combination of the results from at least two studies, it was found that stroke survivors with higher levels of social support (*r* = −0.37, *p* < 0.0001), choosing to face the response (*r* = −0.47, *p* < 0.0001), and having a spouse (*OR* = 0.78, *p* = 0.0255) exhibited a lower risk of distress. Conversely, higher levels of psychological distress were associated with lower income groups (*OR* = 6.69, *p* = 0.0044) and higher levels of disease stigma (*r* = 0.61, *p* < 0.0001), avoidance (*r =* 0.48, *p* < 0.0001) and submission confrontation styles (*r* = 0.61, *p* < 0.0001).

**Table 2:** Meta-analysis results summary of related factors.

Meta-analysis results summary of related factors								
Correlated factors	Studies number	Effect Size of the original study	*I* ^2^	*P* (Q test)	Model	Effect size		*p*-value of the overall effect
						r (95%CI)	OR (95%CI)	
Social support	2	r	0.00%	0.8696	Fixed	−0.37 (−0.47, −0.28)		<0.0001
Stigma	3	r	70.81%	0.0211	Random	0.61 (0.46,0.77)		<0.0001
Medical response								
Face to	3	r	85.43%	0.0004	Random	−0.47 (−0.60, −0.35)		<0.0001
Avoidance	3	r	86.32%	0.0002	Random	0.48 (0.34,0.61)		<0.0001
Submit	3	r	95.48%	<0.0001	Random	0.61 (0.41,0.81)		<0.0001
Age	4	OR	95.20%	<0.0001	Random		0.53 (0.24, 1.17)	0.1174
Gender	6	OR & n	96.30%	<0.0001	Random		0.89 (0.68, 1.17)	0.4132
Educational Level	6	OR & n	99.20%	<0.0001	Random		0.34 (0.05, 2.17)	0.2518
Marriage status	6	OR & n	88.20%	<0.0001	Random		0.78 (0.63, 0.97)	0.0255
Stroke type	3	n	13.90%	0.313	Fixed		0.95 (0.70, 1.28)	0.7286
Low income	3	n	91.50%	<0.0001	Random		6.69 (1.81, 24.69)	0.0044
Living in a town	3	n	66.70%	0.0459	Random		0.69 (0.44, 1.08)	0.1034

### 3.5 Publication bias and clipping methods

The Funnel Plot revealed a significant publication bias with regard to the variables of age, gender, educational level, marital status and stroke type in the aforementioned analyses (Supplementary Figure A.3). Following the application of the trim and fill method, as illustrated in Trim Filling Plots 1 and 2 (Supplementary Figures A.4, A.5), two items were added for age, three items for gender, three items for educational level, two items for marital status, and two items for stroke type. The merger resulted in no appreciable alteration in the overall model effect sizes. The statistically significant marriage status of the model *OR* = 0.73 [95%CI: 0.59–0.90].

### 3.6 Heterogeneity analysis

The heterogeneity of the studies was explored in greater detail as follows: the heterogeneity can be explained after the introduction of the stigma in the number of males (*I*^2^ = 0%, *PQE* = 0.8454). The heterogeneity of the confrontation confronting coping style was largely explained after the introduction of the proportion of high-income people in each study (*I*^2^ = 43.10%, *PQE* = 0.1849). The majority of the heterogeneity associated with the evasive confronting style was explained following the introduction of the proportion of first-time strokes in each study (*I*^2^ = 61.11%, *PQE* = 0.1088). Similarly, the heterogeneity of the yielding coping style could be explained after introducing the proportion of people with ischemic stroke across studies (*I*^2^ = 0.00%, *PQE* = 0.9987). Heterogeneity could be explained after introducing the proportion of people with a spouse as a covariate in the analysis of age in a meta-regression (*I*^2^ = 0.00%, *PQE* = 0.4646). Additionally, heterogeneity of gender could be explained by combining the proportion of low-income people (*I*^2^ = 16.37%, *PQE* = 0.2742). The heterogeneity in marital status can be well explained by the proportion of low-income individuals (*I^2^* = 0.00%, *PQE* = 0.7214). By adding marital status to the merger of low-income entries, heterogeneity could be partially explained (*I*^2^ = 58.69%, *PQE* = 0.1197). Similarly, by adding marital status as a covariate in the merger where the place of residence was town, heterogeneity could be explained (*I*^2^ = 0.00%, *PQE =* 0.7370). In contrast, the observed heterogeneity in education level across studies could not be explained by the data available at this time.

## 4 Discussion

This study makes a contribution to the assessment of the prevalence and factors influencing psychological distress in stroke survivors. The included studies encompass countries and regions from Asia, Africa, Europe, and North America, thereby ensuring a certain level of representativeness. The meta-analysis of the results of the studies revealed a pooled incidence of distress of 51.97%. Furthermore, the results remained consistent when each study was excluded individually using the exclusion method. This indicates that distress after stroke is a prevalent phenomenon across the globe and represents a considerable burden on the mental wellbeing of those who have experienced it.

The incidence of stroke varies significantly according to race and ethnicity (49–51). Despite the disparate assessment instruments utilized by the included studies, the findings of the present study indicate that stroke survivors in China exhibited a relatively elevated prevalence of distress in comparison to their counterparts in the United States. The results were quite different even though three studies used the same assessment tool (33, 42, 45). This is probably due to the fact that only one of the studies was conducted on the American population which means that the trend is limited. Notably, the prevalence of psychological distress among stroke survivors in China appears to be higher than that reported in the United States and other international cohorts. Our meta-analysis yielded a pooled prevalence of 60% (95% CI: 55–65%; *I^2^* = 87%; *p* < 0.01), based on a random-effects model. This may be due to the fact that compared to other countries and regions, China not only has a larger population of patients (3), but also its unique ethnic culture, social structure, and lifestyle may lead to a large number of stroke survivors experiencing psychological maladaptation to stroke events. For example, the family plays an important role in Chinese society. While stroke survivors receive better family support, they often also face more self-perceived burdens.

The available evidence suggests that gender factors may have an impact on the incidence of stroke (52, 53). There is a notable discrepancy in the burden of death and disability from stroke between men and women. By combining the gender factor, it was found that there was no significant correlation between the gender factor and psychological distress. However, the gender factor played a significant role in explaining the heterogeneity in incidence rates across studies, which may suggest that gender has a potential role in the occurrence of distress among stroke survivors. Furthermore, three studies demonstrated a robust positive correlation between stigma and distress, with no notable bias (36, 39, 46). The observed heterogeneity can be attributed to the number of male participants. The analysis of coping strategies was similar, with three studies included in the analysis. These findings may suggest that the role of gender in the occurrence and development of psychological distress is complex and subtle, requiring discussion in conjunction with other factors.

Age is a significant risk factor for stroke occurrence (54, 55). Although it was used to explain the heterogeneity in the incidence of distress, the current study did not identify a statistically significant correlation between age and the dependent variable. This may be attributed to the fact that the majority of the studies presented the mean age of the entire cohort, which precluded their inclusion in the meta-analysis. Only four studies (30, 31, 38, 43) were employed in the age factor meta-analyses, and these may be subject to certain limitations.

It has been demonstrated that social support has a significant impact on the ability of stroke survivors and their families to effectively cope with the social and familial consequences of the condition (56–58). The enhancement of accessible social support may prove an efficacious strategy for the mitigation or prevention of psychiatric distress (59). In this study, five of the included studies (28, 31, 43, 46, 47) addressed the impact of social support factors on psychological distress; however, only two studies (28, 46) reported results of correlation coefficients between social support and distress. The combined analyses indicated a weak negative association between social support and distress. However, no significant heterogeneity or publication bias was identified among the studies. Furthermore, our findings indicate that married survivors may demonstrate lower distress salience. There is a correlation between income level and the incidence of stroke (60, 61). It is estimated that approximately 70% of deaths from stroke and 87% of stroke-related disability occur in low-income and middle-income countries (62). The present study also demonstrated a correlation between income level and the prevalence of psychological distress whereas individuals with low incomes are more likely to exhibit higher psychological distress salience. It also played a significant role in explaining the heterogeneity in the incidence of distress. The observed variation across studies suggests that income and marital status are likely interdependent, with their interactive effects potentially contributing to the heterogeneity of reported mental health outcomes.

Stroke and distress in stroke survivors are strongly associated with educational level (63, 64). Although it has shown a significant correlation with distress in a large number of studies (28–31, 34, 38, 43), we also believe that different levels of education lead to varying cognitive levels, which can affect stroke survivors’ understanding and adaptation to stroke events, thereby influencing the outcomes of psychological distress. In the combined analysis of six studies, the correlation between education level and distress did not show statistical significance. Moreover, there is unexplained heterogeneity among the studies, which makes it difficult to determine whether education level plays a role in the occurrence of distress among stroke survivors.

Psychological distress in stroke survivors is strongly associated with coping strategies. No significant risk of publication bias was observed in any of the three studies. In heterogeneous studies, studies with a higher proportion of more educated survivors among those who chose confrontation exhibited weaker correlations with the occurrence of psychological distress. The study with the stronger correlation with distress among avoidant survivors had a lower proportion of first-time strokes compared with the other two studies. Among compliant survivors, the study with the higher correlation coefficient had a greater proportion of ischemic stroke patients. Based on these results, we hypothesize that the association between confrontational coping and psychological distress may be moderated by educational level. Given the exploratory nature of this observation, these hypotheses require rigorous validation in future research.

### 4.1 Strengths and limitations

To our knowledge, this is the pioneering study to assess the prevalence of psychological distress in stroke survivors and potential influencing factors. To guarantee the robustness and applicability of our findings in clinical practice, we conducted sensitivity analyses on the included clinical control studies. Furthermore, we meticulously selected and included unreplicated trials to reinforce the internal validity of the study results. This enhances the reliability of the study results and their utility in clinical practice.

The findings of our study may provide new ideas for clinical staff to develop effective and safe clinical interventions to reduce the probability of psychological distress in stroke survivors and to control possible influencing factors. There are limitations to this study. First, the sample size was small. In the meta-analysis of related factors, the number of studies finally used for analysis is relatively small due to the transformation of effect sizes and the large number of studies that did not report the relationship between related factors and distress. Furthermore, the outcome effect sizes reported by various studies differed, which further reduced the number of studies included in the analysis. Second, there may be publication bias among the studies, which can have a significant impact on the results. Although the trim-and-fill method only estimated a few articles published in China, there is more reason to consider whether there are more unpublished or under-conducted studies in other countries and regions, and how these studies may have affected the results. A further update of this study may be needed to explore this issue. Furthermore, heterogeneity was observed among the included studies. The heterogeneity was largely attributed to the introduction of covariates through meta-regression of the included studies. However, the heterogeneity among studies still needs to be carefully considered. Furthermore, due to the lack of original research data, we were unable to explore the complex correlations between various influencing factors, which may require further research to confirm these speculations. Finally, geographical distribution may limit external validity, over 90% of the included studies were conducted in China. Although no country restrictions were applied during the search and screening processes, this geographical imbalance may reflect differences in research output and publication/indexing practices. Therefore, the pooled findings should be interpreted with caution in terms of their generalizability to non-Chinese populations.

## 5 Conclusion

Our systematic review and meta-analysis, encompassing 22 studies and 15,370 stroke survivors globally, demonstrates a substantial burden of psychological distress within this population. The findings reveal that mental health outcomes are significantly modulated by a complex interplay of psychosocial determinants, specifically social support, coping strategies, familial structure, and socioeconomic status. These results underscore the urgent necessity for integrating routine psychological screening and tailored psychosocial interventions into post-stroke care pathways to mitigate long-term psychiatric morbidity.

## Data Availability

The original contributions presented in the study are included in the article/Supplementary material, further inquiries can be directed to the corresponding author.
