# Supplementary material for: The burden of psychological distress in stroke survivors: a systematic review and meta-analysis of sociodemographic and behavioral factors
Source: Front Public Health. 2026 Sep 28;14:1930488. doi: 10.3389/fpubh.2026.1930488 (PMC13655148; doi:10.3389/fpubh.2026.1930488)
Supplement: Supplementary file 1 [file Table_1.DOCX]

Supplementary Material

# 1 Supplementary Figures and Tables

1.1 **Supplementary Figures**

**Supplementary Figure A.1 Funnel plot of incidence**

**Supplementary Figure A.2 Sensitivity analysis of incidence**

**Supplementary Figure A.3 Funnel plot of related factors**

**Supplementary Figure A.4 Trim and fill plot 1**

**Supplementary Figure A.5 Trim and fill plot 2**

## 1.2 Supplementary Table

**Supplementary Table 1. A detailed search strategy for English-language database**

## Supplementary Figures

**Supplementary Figure A.1 Funnel plot of incidence**

**Supplementary Figure A.2 Sensitivity analysis of incidence**

**Supplementary Figure A.3 Funnel plot of related factors**

**Supplementary Figure A.4 Trim and fill plot 1**

**Supplementary Figure A.5 Trim and fill plot 2**

## Supplementary Table

**Supplementary Table 1. A detailed search strategy for English-language database**

| #1 | Search: (("Stroke"[Mesh]) OR (Strokes) OR (Cerebrovascular Accident) OR (Cerebrovascular Accidents) OR (Cerebral Stroke) OR (Cerebral Strokes) OR (Stroke, Cerebral) OR (Strokes, Cerebral) OR (Cerebrovascular Apoplexy) OR (Apoplexy, Cerebrovascular) OR (Vascular Accident, Brain) OR (Brain Vascular Accident) OR (Brain Vascular Accidents) OR (Vascular Accidents, Brain) OR (Cerebrovascular Stroke) OR (Cerebrovascular Strokes) OR (Stroke, Cerebrovascular) OR (Strokes, Cerebrovascular) OR (Apoplexy) OR (CVA (Cerebrovascular Accident)) OR (CVAs (Cerebrovascular Accident)) OR (Stroke, Acute) OR (Acute Stroke) OR (Strokes, Acute) OR (Acute Strokes) OR (Cerebrovascular Accident, Acute) OR (Acute Cerebrovascular Accident) OR (Acute Cerebrovascular Accidents) OR (Cerebrovascular Accidents, Acute)) |
| --- | --- |
| #2 | (("Psychological Distress"[Mesh]) OR (Distress, Psychological) OR (Emotional Distress) OR (Distress, Emotional)) |
| #3 | #1 AND #2 |

e.g. PubMed
